# Constitutive Overexpression of the *OsNAS* Gene Family Reveals Single-Gene Strategies for Effective Iron- and Zinc-Biofortification of Rice Endosperm

**DOI:** 10.1371/journal.pone.0024476

**Published:** 2011-09-06

**Authors:** Alexander A. T. Johnson, Bianca Kyriacou, Damien L. Callahan, Lorraine Carruthers, James Stangoulis, Enzo Lombi, Mark Tester

**Affiliations:** 1 School of Botany, The University of Melbourne, Melbourne, Victoria, Australia; 2 Australian Centre for Plant Functional Genomics, University of Adelaide, Glen Osmond, South Australia, Australia; 3 School of Biological Sciences, Flinders University of South Australia, Adelaide, South Australia, Australia; 4 Metabolomics Australia, School of Botany, The University of Melbourne, Melbourne, Victoria, Australia; 5 Centre for Environmental Risk Assessment and Remediation, University of South Australia, Mawson Lakes, South Australia, Australia; United States Department of Agriculture, Agricultural Research Service, United States of America

## Abstract

**Background:**

Rice is the primary source of food for billions of people in developing countries, yet the commonly consumed polished grain contains insufficient levels of the key micronutrients iron (Fe), zinc (Zn) and Vitamin A to meet daily dietary requirements. Experts estimate that a rice-based diet should contain 14.5 µg g^−1^ Fe in endosperm, the main constituent of polished grain, but breeding programs have failed to achieve even half of that value. Transgenic efforts to increase the Fe concentration of rice endosperm include expression of ferritin genes, nicotianamine synthase genes (NAS) or ferritin in conjunction with NAS genes, with results ranging from two-fold increases via single-gene approaches to six-fold increases via multi-gene approaches, yet no approach has reported 14.5 µg g^−1^ Fe in endosperm.

**Methodology/Principal Findings:**

Three populations of rice were generated to constitutively overexpress *OsNAS1*, *OsNAS2* or *OsNAS3*, respectively. Nicotianamine, Fe and Zn concentrations were significantly increased in unpolished grain of all three of the overexpression populations, relative to controls, with the highest concentrations in the *OsNAS2* and *OsNAS3* overexpression populations. Selected lines from each population had at least 10 µg g^−1^ Fe in polished grain and two *OsNAS2* overexpression lines had 14 and 19 µg g^−1^ Fe in polished grain, representing up to four-fold increases in Fe concentration. Two-fold increases of Zn concentration were also observed in the *OsNAS2* population. Synchrotron X-ray fluorescence spectroscopy demonstrated that *OsNAS2* overexpression leads to significant enrichment of Fe and Zn in phosphorus-free regions of rice endosperm.

**Conclusions:**

The *OsNAS* genes, particularly *OsNAS2*, show enormous potential for Fe and Zn biofortification of rice endosperm. The results demonstrate that rice cultivars overexpressing single rice *OsNAS* genes could provide a sustainable and genetically simple solution to Fe and Zn deficiency disorders affecting billions of people throughout the world.

## Introduction

Rice is the primary source of food for roughly half of the world's population yet the polished grain, also known as white rice, contains nutritionally insufficient concentrations of iron (Fe), zinc (Zn) and pro-vitamin A to meet daily requirements in diets based on this staple [1], [2]. Other widely consumed cereals, such as wheat and maize, are also poor sources of several key micronutrients. As a result, micronutrient deficiencies afflict billions of people throughout that world and are particularly prevalent in developing countries where cereals are widely consumed. Fe deficiency affects more than two billion people worldwide, with symptoms ranging from poor mental development and depressed immune function to anaemia, and is the most widespread nutritional deficiency in the world [3]. The development of new cereal varieties containing increased concentrations of Fe and other essential micronutrients, an approach known as biofortification, offers an inexpensive and sustainable solution to the chronic micronutrient malnutrition problems that currently plague people in developing countries.

Rice has the lowest Fe concentration of the cultivated cereal crops and a striking lack of genetic variation for this trait has hindered conventional breeding efforts from increasing its Fe concentration beyond 6 µg g^−1^ in polished grain [4], [5]. To produce polished grain with 14.5 µg g^−1^ Fe, the target concentration that nutritionists have recommended to meet Fe requirements in a rice-based diet, novel sources of genetic diversity for grain Fe concentration are required [6], [7]. Numerous biotechnological strategies have been employed to produce rice with increased concentrations of Fe in endosperm tissues; the principle constituent of polished grain. Grain-specific overexpression of genes encoding ferritin, a Fe storage protein found in plants, animals and bacteria, has been utilized to increase the “sink” for Fe in endosperm [8]. While this approach has resulted in a two-fold increase in endosperm Fe concentration, the relatively modest increases in Fe concentration do not match the 13-fold increase in endosperm ferritin protein levels that often occur via this strategy [9], suggesting that transport of Fe to the endosperm sink is also limiting.

Nicotianamine (NA) is a chelator of transition metals that plays important roles in long- and short-distance transport of metal cations, including Fe^2+^ and Fe^3+^, in higher plants [10], [11]. NA is biosynthesized by trimerization of S-adenosylmethionine, a reaction catalyzed by the NA synthase (NAS) enzymes. Genes encoding NAS are known to be differentially regulated by iron status in a variety of plant species including maize, Arabidopsis, barley and rice [12]–[15], and show strong induction by Fe deficiency. Two of the three rice NAS genes (*OsNAS1* and *OsNAS2*), for instance, show stelar-specific root expression under Fe sufficiency that becomes more ubiquitous throughout root and shoot tissues under Fe deficiency, while a third NAS gene (*OsNAS3*) changes from a primarily shoot-specific expression pattern under Fe sufficiency to more root-specific expression under deficiency [13]. Aside from its role in metal transport in plants, NA is an antihypertensive substance in humans and rice lines with enhanced NA concentration have been developed as potential candidates for the functional food industry [16].

Transgenic approaches to increase NA concentration have often focused on overexpression of exogenous *NAS* genes in plants. Constitutive overexpression of a barley NAS gene, *HvNAS1*, in Arabidopsis and tobacco led to a several-fold increase in seed Fe, Zn and Cu concentration of both species [17]. Similar overexpression of *HvNAS1* in rice led to greatly enhanced NA concentration (15-fold increase over wild type) and 2.3- and 1.5-fold increases in Fe and Zn concentrations of polished grain, respectively [18]. Constitutive expression of an Arabidopsis *NAS* gene, *AtNAS1*, in conjunction with endosperm-specific expression of genes encoding ferritin and phytase, led to a 6.3-fold increase in Fe concentration of rice endosperm [19].

Relatively few studies have been done to overexpress the endogenous rice *NAS* genes (*OsNAS*) in rice. Endosperm-specific overexpression of *OsNAS1* resulted in polished grain with significantly increased concentrations of NA and Zn and, although Fe concentration was not increased by this strategy, the bioavailability of Fe was double that of controls as measured by ferritin synthesis in Caco-2 cells [20]. Recently, activation tagged lines of rice with increased expression of *OsNAS2* and *OsNAS3* were identified and characterized [21], [22]. An *OsNAS2* activation tagged line had 20-fold more NA and 2.7-fold more Zn in polished grain, while two *OsNAS3* activation tagged lines had up to nine-fold more NA, 2.6-fold more Fe and 2.2-fold more Zn in polished grain. Most significantly, polished grain from an *OsNAS3* activation tagged line reversed signs of Fe-deficiency when fed to anemic mice [21].

The overall aim of this study was to constitutively overexpress all three members of the *OsNAS* gene family, individually, to assess their utility for Fe biofortification of polished rice grain via a single-transgene approach. Characterization of more than 90 independent transgenic lines overexpressing these genes revealed that all three *OsNAS* genes increase not only Fe, but also Zn concentrations in unpolished and polished grain when expressed constitutively and those increases are positively correlated with NA concentration. One member of the *OsNAS* gene family, *OsNAS2*, was particularly effective at increasing Fe and Zn concentrations in rice endosperm and this increase was mapped in unprecedented detail using synchrotron X-ray fluorescence spectroscopy (µ-XRF).

## Results

### Construction of three rice populations overexpressing *OsNAS1*, *OsNAS2* and *OsNAS3*

**Figure 1:**
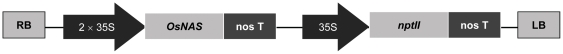
Schematic representation of the T-DNAs used for constitutive overexpression of the three *OsNAS* genes. RB, right border; 2 × 35S, dual CaMV 35S promoter; *OsNAS*, coding sequence of *OsNAS1* (999 bp), *OsNAS2* (981 bp) or *OsNAS3* (1032 bp); nos T, nopaline synthase terminator; 35S, CaMV 35S promoter; nptII, *neomycin phosphotransferase II*; LB, left border.

The 0.7 kb dual CaMV 35S promoter contained in the pMDC vector system [23] was used to drive constitutive expression of the *OsNAS1* (LOC_Os03g19427), *OsNAS2* (LOC_Os03g19420) and *OsNAS3* (LOC_Os07g48980) coding sequences in rice. The T-DNA region of the binary vectors used for transformations also contained the selectable marker gene *neomycin phosphotransferase II* that detoxifies aminoglycoside antibiotics such as geneticin (G418) and kanamycin (Figure 1). Embryogenic callus of japonica rice cultivar Nipponbare was used for *Agrobacterium*-mediated transformation of the binary vectors containing the three different *OsNAS* coding sequences. The production of 30 independent transgenic lines carrying the *OsNAS1* overexpression vector, designated the OE-*OsNAS1* population, 39 independent transgenic lines carrying the *OsNAS2* overexpression vector, designated the OE-*OsNAS2* population, and 24 independent transgenic lines carrying the *OsNAS3* overexpression vector, designated the OE-*OsNAS3* population, was confirmed by resistance to geneticin in T_0_ and T_1_ plants, PCR and Southern blot analysis (data not shown).

### Constitutive overexpression of the *OsNAS* genes leads to increased Fe and Zn concentrations in unpolished and polished grain

**Figure 2:**
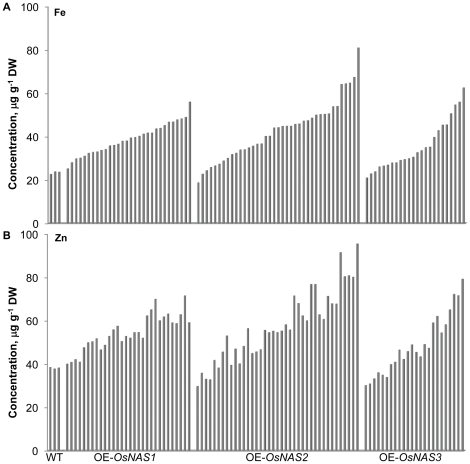
Fe (A) and Zn (B) concentrations in unpolished grain of wild type and transgenic rice. WT, three wild type lines of rice; OE-*OsNAS1*, 30 independent transgenic lines overexpressing *OsNAS1*; OE-*OsNAS2*, 39 independent transgenic lines overexpressing *OsNAS2*; OE-*OsNAS3*, 24 independent transgenic lines overexpressing *OsNAS3*. Unpolished grain was analyzed by ICP-OES to determine Fe and Zn concentrations. The three populations of overexpression lines are sorted in order from lowest to highest Fe concentration in panels A and B.

Because nicotianamine is known to chelate and mobilize a variety of metal cations including Fe, Zn, Mn, Cu and Ni in plants [24], [11] we employed inductively coupled plasma optical emission spectrometry (ICP-OES) to characterize the elemental composition of unpolished T_1_ grain harvested from a single season of growth of the three transgenic rice populations in the glasshouse. Two metals in particular, Fe and Zn, were several-fold higher in unpolished grain of the OE-*OsNAS* population, relative to WT grain (Figure 2), while Mn, Cu and Ni did not show significant differences from WT grain (Table S1).

Unpolished grain Fe concentrations ranged from 25 to 56 µg g^−1^ dry weight (DW) in the OE-*OsNAS1* population, 19 to 81 µg g^−1^ DW in the OE-*OsNAS2* population and 21 to 63 µg g^−1^ DW in the OE-*OsNAS3* population, representing up to 2.4-fold, 3.5-fold and 2.7-fold increases, respectively, over wild type (WT) in each population (Figure 2A). Unpolished grain Zn concentrations ranged from 40 to 59 µg g^−1^ dry weight (DW) in the OE-*OsNAS1* population, 30 to 95 µg g^−1^ DW in the OE-*OsNAS2* population and 30 to 79 µg g^−1^ DW in the OE-*OsNAS3* population, representing up to 1.9-fold, 2.5-fold and 2.1-fold increases, respectively, over wild type (WT) in each population (Figure 2B). Furthermore, Fe and Zn concentrations of unpolished grain were highly correlated in transgenic lines (r = 0.83, 0.94 and 0.97 for the OE-*OsNAS1*, OE-*OsNAS2* and OE-*OsNAS3* populations, respectively) which accounts for the similar Fe and Zn profiles in Figure 2A and 2B (genotype order is the same in both panels).

Polished grain was produced from selected transgenic lines using a modified non-contaminating Kett Mill. One transgenic line overexpressing *OsNAS1* (OE-*OsNAS1S*), two independent transgenic lines overexpressing O*sNAS2* (OE-*OsNAS2B* and OE-*OsNAS2J*) and one transgenic line overexpressing *OsNAS3* (OE-*OsNAS3B*) were selected for this analysis based on large numbers of available grain to mill and Fe concentrations that fell within the upper 20% of each overexpression population. WT had a Fe concentration of 4.5 µg g^−1^ DW in the polished grain, representing approximately 20% of Fe concentration in the unpolished grain (Table 1). The OE*-OsNAS1S* and OE*-OsNAS3B* lines had Fe concentrations of approximately 10 µg g^−1^ DW in polished grain, representing (like WT) 20% of Fe concentrations in the unpolished grain and a two-fold increase over WT concentration. The two *OsNAS2* overexpression lines (OE-*OsNAS2B* and OE-*OsNAS2J*) had Fe concentrations of 14–19 µg g^−1^ DW in polished grain, representing 26–30% of Fe concentrations in the unpolished grain and up to a 4.2-fold increase over WT concentration. Zinc concentrations were also increased in polished grain of the transgenic lines. The OE*-OsNAS1S* and OE*-OsNAS3B* lines had approximately 49 µg g^−1^ DW Zn in polished grain, representing a 1.4-fold increase over WT concentration. The OE-*OsNAS2B* and OE-*OsNAS2J* lines had 52–76 µg g^−1^ DW Zn in polished grain, representing up to a 2.2-fold increase over WT concentration. As with unpolished grain, the Fe and Zn concentrations in polished grain were highly correlated (r = 0.94).

**Table 1:** Concentrations of Fe and Zn in unpolished and polished grain of WT and transgenic rice.

Genotype	Unpolished (µg g^−1^)		Polished (µg g^−1^)		% Fe in polished	% Zn in polished
	Fe	Zn	Fe	Zn		
**WT**	22	42	4.5	34	21	81
**OE-** ***OsNAS1S***	47	63	9.7	48	21	76
**OE-** ***OsNAS2B***	64	91	19	76	30	84
**OE-** ***OsNAS2J***	54	68	14	52	26	77
**OE-** ***OsNAS3B***	51	65	9.9	49	19	75

Grain samples from WT, one transgenic line overexpressing *OsNAS1* (OE-*OsNAS1S*), two independent transgenic lines overexpressing O*sNAS2* (OE-*OsNAS2B* and OE-*OsNAS2J*) and one transgenic line overexpressing *OsNAS3* (OE-*OsNAS3B*) were analyzed by ICP-OES. The percentage of Fe and Zn concentration in polished grain, relative to unpolished grain concentration, is presented in the last two columns of the table.

### Constitutive overexpression of the *OsNAS* genes leads to increased NA concentrations in unpolished grain that are positively correlated with Fe and Zn concentration

Liquid chromatography-mass spectrometry (LC-MS) was employed to determine if *OsNAS* overexpression leads to significantly increased NA concentration of the grain. Single-insert transgenic lines were selected for nicotianamine quantification experiments to ensure that null segregant lines (lines that have the lost the overexpression vector due to meiotic segregation) were produced as additional controls to WT. Three sibling T_1_ lines, comprising two transgenic lines and one null segregant line, were derived from a single T_0_ parental line within each of the three OE-*OsNAS* populations and grown to maturity in a growth room to yield T_2_ grain. The T_0_ parents of the OE-*OsNAS1*, OE-*OsNAS2* and OE-*OsNAS3* T_1_ siblings had unpolished grain Fe concentrations of 56, 64 and 51 µg g^−1^ DW, respectively, and unpolished grain Zn concentrations of 59, 80 and 65 µg g^−1^ DW, respectively. In addition to nicotianamine quantification by LC-MS, the Fe and Zn concentrations of T_2_ grain were determined by ICP-OES.

**Figure 3:**
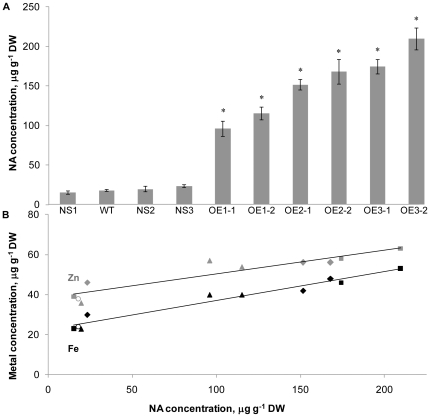
Fe and Zn concentrations in unpolished grain are positively correlated with nicotianamine (NA) concentration. (A) NS, null segregant lines of rice; WT, wild type line of rice; OE, overexpression lines of rice. Three sibling T_1_ lines, consisting of two OE lines and one NS line, were obtained from a single-insert T_0_ mother line in each of the three *OsNAS* overexpression populations. The three *OsNAS1* sibling lines are labeled OE1-1, OE1-2 and NS1; the three *OsNAS2* sibling lines are labeled OE2-1, OE2-2 and NS2; the three *OsNAS3* sibling lines are labeled OE3-1, OE3-2 and NS3. Unpolished grain was analyzed by LC-MS to determine nicotianamine concentration (mean ± SE, n = 4). Significant differences from WT were determined by Student's *t* test and are indicated by asterisks (P<0.05). (B) Statistically significant positive correlations were found between unpolished grain NA concentration and Fe (black shapes; r = 0.9769 and p<0.01) and Zn (gray shapes; r = 0.9288 and P<0.01) concentrations for the ten genotypes described in panel A. The six OE1, OE2 and OE3 sibling lines are represented by triangles, diamonds and squares, respectively. The three NS lines appear just next to the WT line (represented by circles) on the scatter chart.

The unpolished grain NA concentration was 18 µg g^−1^ DW for WT and did not differ significantly from unpolished grain NA concentrations of null segregant (NS) lines (Figure 3A). By contrast, unpolished grain NA concentrations ranged from 96 to 115 µg g^−1^ DW in the OE-*OsNAS1* sibling lines, 152 to 168 µg g^−1^ DW in the OE-*OsNAS2* sibling lines and 174 to 210 µg g^−1^ DW in the OE-*OsNAS3* sibling lines, representing up to 6.4-fold, 9.3-fold and 11.7-fold increases, respectively, over wild type (WT) concentrations of NA. Figure 3B demonstrates the statistically significant, positive correlation that was found between unpolished grain NA concentration and Fe and Zn concentration for the ten genotypes utilized in this experiment. While the OE-*OsNAS3* siblings produced T_2_ grain with the same Fe and Zn concentrations as the T_0_ parent, the Fe concentration of T_2_ grain from the OE-*OsNAS1* siblings and Fe and Zn concentrations of T_2_ grain from the OE-*OsNAS2* siblings were lower than that of the T_0_ parents (approximately 10–15 µg g^−1^ DW lower). These results indicate that grain Fe and Zn concentrations of certain transgenic events are more consistent than others across varying environments (in this case glasshouse vs. growth room) and that all events should be evaluated over several sexual cycles and under differing conditions including the field.

### µ-XRF elemental maps reveal significant increases in Fe and Zn accumulation in specific tissues of *OE-OsNAS2* grain

**Figure 4:**
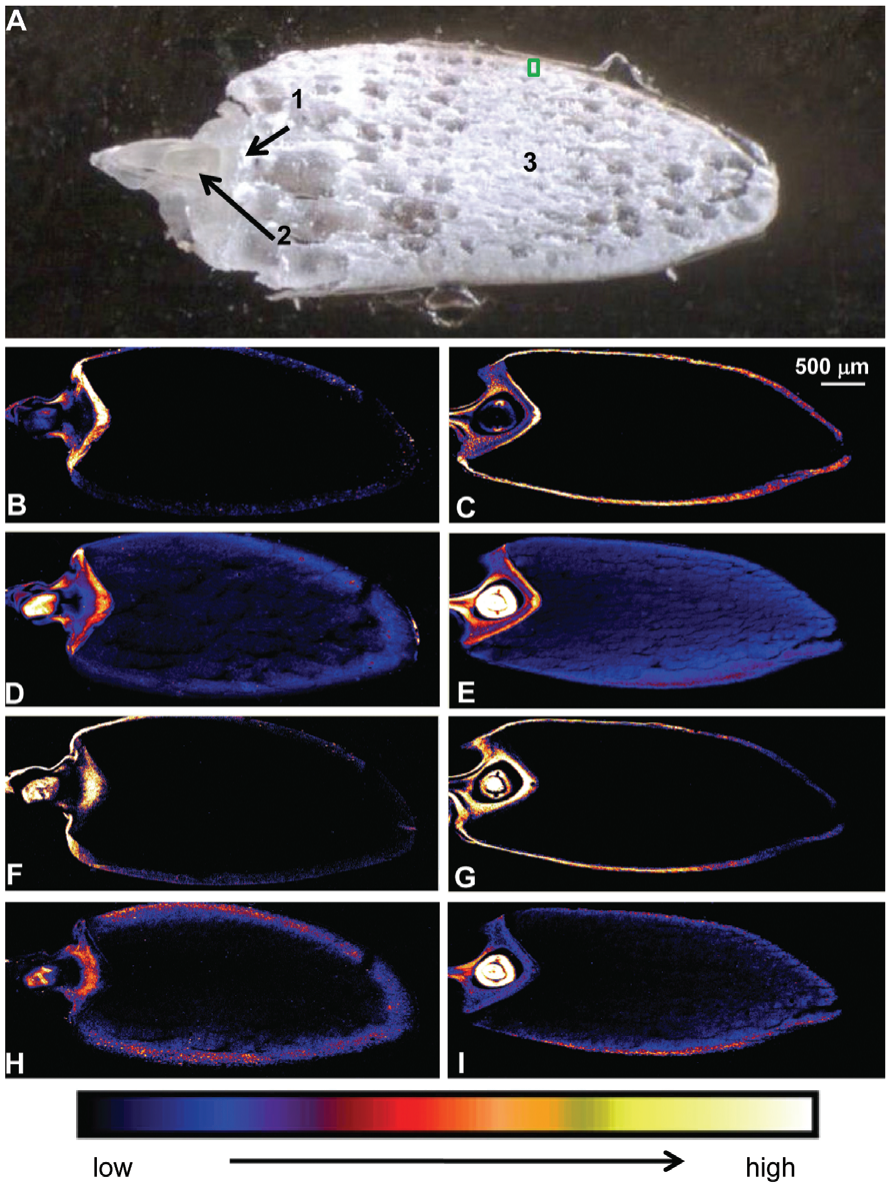
µ-XRF elemental maps of WT and OE-*OsNAS2A* longitudinal grain sections. WT grain had 23 µg g^−1^ DW Fe and 38 µg g^−1^ DW Zn while OE-*OsNAS2A* grain had 64 µg g^−1^ DW Fe and 80 µg g^−1^ DW Zn, as determined by ICP-OES. (A) Light microscopy photo of a representative grain section with numbers indicating the location of scutellum (1), embryo (2) and endosperm (3); the green box represents the area used to obtain the line scans in Figures 5–6. (B–I) Elemental maps of Fe distribution in WT (B) and OE-*OsNAS2A* (C) grain; Zn distribution in WT (D) and OE-*OsNAS2A* (E) grain; Mn distribution in WT (F) and OE-*OsNAS2A* (G) grain; Cu distribution in WT (H) and OE-*OsNAS2A* (I) grain. The colour scale represents different elemental concentrations, with black and white corresponding to the lowest and highest concentrations, respectively.

Synchrotron X-ray fluorescence spectroscopy (µ-XRF) was used to generate elemental distribution maps of several NA-related cations (Fe, Zn, Mn and Cu) in two longitudinal sections each of WT and OE-*OsNAS2A* grain (four sets of elemental distribution maps total). Elemental distribution in the two longitudinal sections of each grain type was very similar and therefore only one set of images for each grain type is presented in Figure 4.

The maps of Fe distribution in WT and OE-*OsNAS2A* grain (Figures 4B and C, respectively) show a striking lack of detectable Fe signal in large portions of the endosperm. In WT grain, the highest signal occurs in scutellum and outer regions of the embryo while a very low Fe signal is detected in the outermost layers of the endosperm and the single-layered aleurone that surrounds the endosperm. No signal is detected from the inner layers of the endosperm. The OE-*OsNAS2A* grain, by contrast, has a high Fe signal in the outer endosperm and aleurone layers in addition to high aleurone and embryo signals. The inner layers of endosperm, as with WT grain, have no signal.

The maps of Zn distribution in WT and OE-*OsNAS2A* grain (Figures 4D and E, respectively) depict a radically different distribution of this element compared to Fe. In WT grain, the highest signal is observed inside the embryo (likely corresponding to the plumule) while the scutellum and outer embryo has intermediate signals. A low signal is detected in a thick band comprising many outer layers of endosperm and the single-layered aleurone that surrounds the endosperm. Unlike Fe, the Zn signal extends (albeit at very low levels) throughout the endosperm of WT grain. In OE-*OsNAS2A* grain a similar pattern of Zn distribution is observed, however, the signal in the embryo and throughout endosperm tissues is considerably higher. In the outermost layers of the endosperm and the single-layered aleurone, the signal borders on intermediate signal intensity.

The maps of Mn and Cu in WT and OE-*OsNAS2A* grain (Figures 4F and G, 4H and I, respectively) show that these metals, like Fe, have no signal in much of the endosperm. The Mn signal is higher in the outermost layers of the endosperm and the single-layered aleurone of OE-*OsNAS2A* compared to WT, and this may be explained by the slightly higher Mn concentration detected by ICP-OES for the OE-*OsNAS2A* grain relative to WT (14 vs. 11 µg g^−1^ DW Mn, respectively). The Cu signal is higher in the embryo of OE-*OsNAS2A* compared to WT, and this may be explained by the slightly higher Cu concentration detected by ICP-OES for the OE-*OsNAS2A* grain relative to WT (9 vs. 7 µg g^−1^ DW Cu, respectively).

### XRF line scans reveal significantly more Fe and Zn, and larger Fe:Zn ratios, in aleurone, subaleurone and endosperm tissues of *OE-OsNAS2* grain

**Figure 5:**
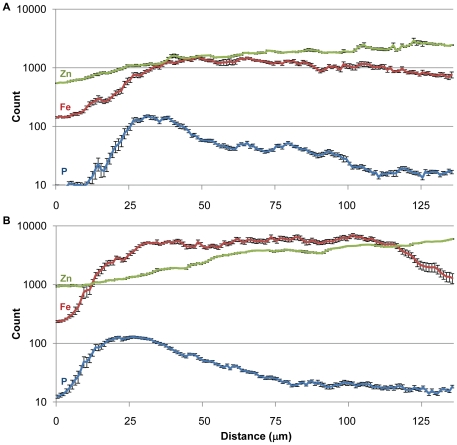
Line scans for P, Fe and Zn in WT (A) and OE-*OsNAS2A* (B) grain. Line scans begin on the outer margin of grain and continue 135 µm towards the endosperm; data is displayed as average count (mean ± SE, n = 23). Counts are plotted on logarithmic scale in the y-axis to account for the low P counts (blue) relative to Fe (red) and Zn (green). As the rice grain contains a single aleurone layer that is rich in phosphorus (P, primarily in the form of phytic acid) while endosperm contains little P, 1–50 µm was assigned to the aleurone layer, 51–90 µm to the subaleurone layer and 91–135 µm to the endosperm.

Two 135 µm line scans across the grain, away from the embryo region, are reported for WT and OE-*OsNAS2A* in Figure 5. These line scans were obtained by laterally averaging a box (represented by a square in Figure 4A) with a width of 23 pixels so that each count represents the average of 23 line scans of the grain. The rice grain contains a single aleurone layer that is rich in phosphorus (P, primarily in the form of phytic acid) while starchy endosperm tissues contain only trace P [25], [26]. The P distribution, which had highly similar counts and profile in both WT and OE-*OsNAS2A* grain, was thereby used to assign regions of the line scan to aleurone, subaleurone and endosperm layers of grain. As the rectangular-shaped aleurone cells of rice endosperm are approximately 25–30 µm in length [27], we conservatively assigned the first 50 µm of the line scan to the aleurone layer (the additional 20 µm accounting for the pericarp, seed coat and nucellus that precede the aleurone layer). Consistent with the phytic acid-enriched aleurone layer, the average P counts for both WT and OE-*OsNAS2A* grain were by far the highest in this 50 µm section (70 and 81 counts, respectively). P counts began to rapidly drop off after 50 µm and we assigned 51–90 µm to the subaleurone layer and 91–135 µm to the endosperm (assuming average cell lengths of 40 µm in these two layers). Average P counts for WT and OE-*OsNAS2A* grain were roughly halved in the subaleurone layer (44 and 30 counts, respectively) and nearly background levels in the endosperm layer (20 and 18 counts, respectively).

The line scan of WT grain (Figure 5A) shows that Fe and Zn counts increase rapidly through the aleurone layer and are nearly equal towards the start of the subaleurone layer (∼1550 counts for both Fe and Zn at 50 µM). The Fe count begins to drop off beyond this point while Zn continues to climb for most of the subaleurone and endosperm layers before leveling off in the last 10 µm of the scan. The results demonstrate that while the aleurone layer has significant amounts of Fe and Zn, the subaleurone layer and endosperm layers, combined, have higher amounts of both of these metals. The results also show that there is more Zn relative to Fe for nearly all of the scanned region, leading to Fe:Zn signal ratios of <1 for the aleurone, subaleurone and endosperm layers (Table 2).

**Table 2:** Average counts of Fe and Zn in aleurone, subaleurone and endosperm layers of WT and OE-*OsNAS2A* grain.

Genotype	Aleurone			Subaleurone			Endosperm		
	Fe	Zn	Ratio	Fe	Zn	Ratio	Fe	Zn	Ratio
**WT**	715	1055	0.68	1252	1779	0.70	935	2236	0.42
**OE-** ***OsNAS2A***	3194	1460	2.19	5472	3512	1.56	4268	4757	0.90

The Fe:Zn signal ratio is presented for each of the three layers.

**Figure 6:**
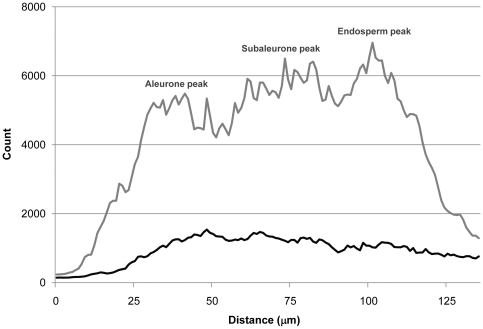
Line scans for Fe in WT and OE-*OsNAS2A* grain. Line scans begin on the outer margin of grain and continue 135 µm towards the endosperm; average WT counts indicated by the black line and average OE-*OsNAS2* counts indicated by the gray line (mean, n = 23). The location of the successively higher Fe peaks in aleurone, subaleurone and endosperm regions of OE-*OsNAS2A* grain is indicated on the figure.

The line scan of OE-*OsNAS2A* grain (Figure 5B) shows that Fe and Zn counts increase rapidly through the aleurone layer but at a much steeper slope for Fe, so that Fe counts surpass Zn counts early in the aleurone layer. The Fe count shows a first peak in the aleurone layer (5482 counts at 41 µm), similar to WT. Unlike the WT grain, however, the OE-*OsNAS2A* grain has two, successively higher, Fe peak regions in the subaleurone (6498 counts at 81 µm) and endosperm (6958 counts at 101 µm) layers. The Fe count begins to drop off beyond this point, most rapidly in the final 20 µm of the scan. The Fe counts in WT and OE-*OsNAS2A* grain are plotted on linear scale in Figure 6 to clearly visualize the differences in Fe quantity and distribution between the two genotypes.

The Fe count is approximately 4.4-fold higher in the aleurone, subaleurone and endosperm layers of OE-*OsNAS2A* grain relative to WT (Table 2). The Zn count in OE-*OsNAS2A* grain rises progressively through the scan, but does not surpass the Fe count until the final 20 µm of the scan. The Zn count is 1.4-fold higher in the aleurone layer and two-fold higher in the subaleurone and endosperm layers of OE-*OsNAS2A* grain relative to WT. These fold increases for Fe and Zn count are remarkably consistent with the 4.2-fold and 2.2-fold increases of Fe and Zn concentration, respectively, that were reported for polished OE-*OsNAS2B* grain, relative to WT, as determined by ICP-OES (Table 1). Because Fe counts are higher than Zn counts for most of the line scan, Fe:Zn signal ratios are much larger for OE-*OsNAS2A* and >1 for both the aleurone and subaleurone layers (Table 2).

## Discussion

The results of this study demonstrate significantly increased Fe and Zn concentrations in rice endosperm as a result of constitutive overexpression of single *OsNAS* genes. Within each of the three transgenic rice populations – OE-*OsNAS1*, OE-*OsNAS2* and OE-*OsNAS3* – lines were identified with at least 2- and 1.5-fold increases in Fe and Zn concentrations, respectively, of unpolished grain. Large differences, however, were observed regarding the upper limits of Fe and Zn enrichment that were found in each population (Figure 1). The OE-*OsNAS1* and OE-*OsNAS2* populations differed most significantly from each other, with the highest Fe-containing OE-*OsNAS1* line (56 µg g^−1^ DW) representing only 70% of the unpolished grain Fe concentration of the highest OE-*OsNAS2* line (81 µg g^−1^ DW). In fact, the five highest Fe-containing OE-*OsNAS2* lines had more than 60 µg g^−1^ DW Fe in unpolished grain. The observed differences between the OE-Os*NAS1* and OE-*OsNAS2* populations are surprising considering that the coding sequences of *OsNAS1* and *OsNAS2* share 87% identity and the first 233 amino acids of the two, roughly 330 aa long enzymes, are identical. The OsNAS1 and OsNAS2 enzymes may show differential activity in the synthesis of NA, or overexpression of the *OsNAS1* and *OsNAS2* coding sequences may cause significant, as yet unknown, pleiotropic effects on nutrient transport processes in rice. Studies regarding both of these possibilities are now underway.

While only a subset of lines were milled to produce polished grain, results obtained with the two OE-*OsNAS2* lines revealed some of the highest Fe concentrations that have been reported for rice endosperm. OE-*OsNAS2B* and OE-*OsNAS2J* had Fe concentrations of 19 and 14 µg g^−1^ DW, respectively, in rice endosperm (Table 1). These concentrations are 4.2- and 3-fold higher, respectively, than the Fe concentration observed for WT polished grain (4.5 µg g^−1^ DW) and represent the first time that rice lines have been reported with Fe concentrations at or above the 14.5 µg g^−1^ DW threshold recommended for a Fe-biofortified rice diet [6]. Zn concentrations of the two OE-*OsNAS2* lines were also 1.5- to 2.2-fold higher than WT polished grain. The increased Fe concentrations of polished OE-*OsNAS2* grain appear due to not only higher metal concentration in unpolished grain, but also reduced losses of Fe during milling of the grain. While Fe concentrations of WT, OE-*OsNAS*1 and OE-*OsNAS3* polished grain represented 20% of unpolished grain Fe concentrations, Fe concentrations of the two OE-*OsNAS2* polished grain samples represented 26–30% of unpolished grain Fe concentration (Table 1). These results suggest that Fe penetrates further into endosperm tissues of the OE-*OSNAS2* grain relative to WT, however, more overexpression lines within each of the three populations require similar characterization to determine whether this trait is specific to only the OE-*OsNAS2* population. The OE-*OsNAS* overexpression lines should also be evaluated under field conditions to determine the stability of the high-Fe trait under different environments where Fe may be more limiting. Field trials will also enable agronomic performance of the lines to be accurately assessed.

The positive correlations between Fe and Zn concentrations in both unpolished and polished grain of the three OE-*OsNAS* populations provided strong evidence that a common mechanism – most likely NA – was responsible for transporting these micronutrient metals into the grain. The LC-MS experiments with segregating T_1_ lines confirmed this hypothesis and showed that NA concentrations in unpolished grain were 6.4- to 11.7-fold higher in the *OsNAS* overexpression progeny relative to WT and null segregant lines. The NA concentration that we calculated for WT unpolished grain using the LC-MS method – 18 µg g^−1^ DW – is very close to published values of 21.2 µg g^−1^ DW for unpolished rice grain [28] and gave us confidence that our analytical technique was accurate and representative of actual NA concentrations. The fact that null segregant lines did not differ significantly from WT with respect to unpolished grain NA concentration, while all of the transgenic progeny had significantly higher concentrations, provided conclusive evidence that the OE-*OsNAS* constructs were responsible for the increases in NA concentration (Figure 3A). Additionally, these experiments demonstrated that the OE-*OsNAS* constructs of single-insert lines were transmitted to progeny lines in typical Mendelian fashion and stably expressed in progeny. The statistically significant, positive correlations between NA concentration and Fe and Zn concentrations (r = 0.9769 and 0.9288, respectively) demonstrated that increased NA concentrations of unpolished grain were not only higher in the *OsNAS* transgenic progeny, but also indicative of Fe and Zn concentration (Figure 3B). NA concentration can therefore be considered a major regulator of Fe and Zn concentrations in rice grain, and NA may very well be a limiting factor in the accumulation of Fe and Zn in WT rice grain.

To further explore the distributions of Fe, Zn and other metal cations (that are known to chelate with NA) in rice grain, we employed synchrotron X-ray fluorescence spectroscopy (µ-XRF) to generate detailed elemental maps of WT and OE-*OsNAS2* longitudinal grain sections. A single-insert transgenic line from the OE-*OsNAS2* population, OE-*OsNAS2A*, with high levels of Fe and Zn (64 and 80 µg g^−1^ DW, respectively) was selected as a comparison to WT grain. The elemental maps of Fe and Zn in WT grain (Figure 4B and D) are in agreement with what we know about the distribution of these two metals in cereal grain – namely that Zn has a higher concentration and more broad distribution profile in the grain compared to Fe and, away from the embryo region, is not limited to outer layers of endosperm and the aleurone. The abundance of Zn in the central portion of the embryo, most likely in the plumule, has been observed in similar µ-XRF studies of barley grain [29]. The complete absence of Fe signal from much of the endosperm, as opposed to Zn which extends (faintly) throughout the endosperm, demonstrates why polishing of rice grain causes much greater losses of Fe compared to Zn and is in line with previous findings [30]. When maps of WT grain were compared to those of OE-*OsNAS2A* grain, one of the most striking differences was in Fe distribution (Figure 4B and C). Whereas WT had very low signals of Fe in the outer endosperm and aleurone layer of the grain, OE-*OsNAS2A* grain had intermediate to high signals in the same position. Another major difference between WT and OE-*OsNAS2A* grain concerned the overall higher signals for Zn throughout the transgenic grain. Although the Zn distribution pattern did not appear altered in OE-*OsNAS2A* grain, the signal intensity was considerably higher. The small differences observed in Mn and Cu intensity between the two grain types were likely due to small (2–3 µg g^−1^ DW) increases of those elements in the embryo and/or aleurone layer of OE-*OsNAS2A* grain.

Line scans allowed us to focus in on the outer region of the grain, away from the embryo region (the area highlighted, as an example, in green in Figure 4A), where the large differences for Fe signal intensity had been observed between WT and OE-*OsNAS2A* grain. Of crucial importance to this experiment was the ability to detect phosphorus (P) as a direct indicator of the aleurone layer. The aleurone cells of cereals accumulate high levels of phosphorus-containing phytic acid (PA), which normally occurs as a mixed salt of potassium (K), magnesium (Mg), calcium (Ca), Fe and Zn in the cells [31]. The primary function of PA is to provide storage of phosphorus and minerals for germinating seeds. The consequences of PA binding to minerals and micronutrients such as Fe and Zn, however, are undesirable from a nutritional point of view. PA is a strong inhibitor of mineral and micronutrient absorption and is reported to inhibit Fe, Zn, Ca and manganese (Mn) absorption in humans [32]. It is thought that mineral binding to PA forms an insoluble complex that precipitates, thereby rendering the mineral unavailable to human intestinal absorption. By detecting P in our line scans, we were able to not only distinguish aleurone cells from starchy endosperm (which has only trace levels of P), but also accurately determine if Fe and Zn were localized to a region where they were likely to be bound by PA (and therefore unlikely to be bioavailable).

The line scans of WT grain showed the highest counts of Fe towards the aleurone/subaleurone junction, after which Fe counts began to slowly decline through the subaleurone and endosperm layers (Figure 5A). Zn counts, on the other hand, steadily increased through the aleurone, subaleurone and endosperm layers, so that the highest Zn counts were detected in the endosperm. Because Zn count was higher than Fe for most of the scan, the Fe:Zn signal ratio in all three layers was <1 (Table 2). The line scan of WT grain provides novel insights into the distribution of Fe in the outer layers of rice grain. While significant quantities of Fe and Zn are localized in the aleurone cells, and therefore likely bound to PA, the subaleurone and endosperm contain substantial quantities of Fe and Zn that are likely to be bioavailable; particularly in the endosperm region where only trace P was detected. What molecule(s) the micronutrient metals are chelated to in this region remains unknown.

The line scans of OE-*OsNAS2A* grain revealed a radically different distribution and quantity of Fe and Zn in all three layers (Figure 5B). A major difference was apparent in the relative amounts of Fe and Zn, with more Fe than Zn counts detected in most of the scanned region. This is essentially the reverse of what was seen in WT grain, and is reflected in Fe:Zn signal ratios of >1 for aleurone and subaleurone cells, and close to 1 in the endosperm, for OE-*OsNAS2A* grain (Table 2). Furthermore, the Fe count did not tail off after a peak in the subaleurone layer, rather, it continued to peak at progressively higher levels in the subaleurone and endosperm layers. In fact, the highest count of Fe in OE-*OsNAS2A* grain (6958 at 101 µm) occurred in a region of endosperm where only trace counts of P (21) were detected, thereby indicating that most of the Fe could not be complexed with PA and may be readily bioavailable. To better visualize the distribution of Fe in OE-*OsNAS2A* grain, and how it differs from that of WT, the Fe counts for both grain types were plotted on linear scale in Figure 6. Increased Fe concentrations in the aleurone, subaleurone and endosperm layers of OE-*OsNAS2A* grain, relative to WT, are readily apparent in this chart and, furthermore, the enrichment of Fe in the endosperm layer of transgenic grain is clear. The Zn counts in OE-*OsNAS2A* grain were also significantly higher than those of WT grain, but followed a similar trend to that of WT by gradually increasing towards the endosperm.

It is tempting to speculate that the increased concentrations of Fe and Zn in aleurone, subaleurone and endosperm layers of OE-*OsNAS2A* grain are present as complexes with NA. NA is known to have high binding affinities for Fe and Zn at alkaline pH, while the Fe^2+^NA complex in particular demonstrates unusually high kinetic stability that does not show autoxidation at physiological pH ranges [11]. Preferential binding of NA to Fe^2+^ as it is transported to the grain through phloem tissues, and high stability of the Fe^2+^NA complex within the grain, could explain why the trend of Zn>Fe counts in WT grain is reversed in OE-*OsNAS2A* grain. The hypothesis that Fe and Zn are bound to NA is bolstered by recent analyses of grain from previously mentioned *OsNAS3* activation tagged line of rice which contains nine-fold more NA in the grain [21]. Whereas WT and the *OsNAS3* activation tagged grain did not differ with regards to the amount of PA-bound Fe, the *OsNAS3* activation tagged grain had seven-fold more Fe bound to a low molecular weight mass compound that is likely to be NA. A similar result was found in grain of the *OsNAS2* activation tagged line with regards to Zn [22]. We are currently using X-ray Absorption Near Edge Structure (XANES) to identify compounds that bind to Fe and Zn in the endosperm of WT and OE-*OsNAS2A* grain. XANES should also yield speciation information for Fe (Fe^2+^ vs. Fe^3+^), and we expect the OE-*OsNAS2A* grain to have more Fe^2+^ relative to Fe^3+^ due to preferential chelation of Fe^2+^ by NA under aerobic conditions [11].

More than 2 billion people are currently afflicted by iron deficiency, a serious nutritional problem that has been exacerbated by high dependences on nutrient poor cereal crops in many developing countries of the world. Billions also suffer from equally devastating micronutrient disorders such as Zn and Vitamin A deficiency. Worryingly, micronutrient malnutrition problems may become even more prevalent as the Earth's atmospheric concentration of carbon dioxide (CO_2_) continues to rise. Many studies have shown that carbon enrichment, while increasing productivity of many crops, also causes significant decreases in the concentration of key micronutrients such as Fe and Zn [33]. In light of these results it is imperative that conventional breeding and biotechnology are exploited to the fullest extent to increase nutritional composition of the world's major food staples. Using constitutive overexpression of single members of the *OsNAS* gene family, we have produced biofortified rice lines that contain significantly enhanced Fe and Zn concentrations in polished grain. The use of rice genes to increase the micronutrient concentration of rice shows that cisgenic plants could be developed using similar technology [34]. Most importantly, the Fe concentrations detected in particular *OsNAS2* overexpressing lines meet or surpass the target concentration for Fe biofortification of rice endosperm. The enhanced Fe concentrations are preferentially located in areas of the rice grain where they are unlikely to be bound by phytic acid and therefore likely to be bioavailable in human diets.

## Materials and Methods

### Plant growth conditions

*Oryza sativa* ssp. *japonica* cv. Nipponbare was used for all experiments. Seeds were germinated on moist filter paper for one week before transfer to 15 cm (1 L capacity) containers of University of California (UC) potting mix in a glasshouse or growth room maintained at 28°C day, 24°C night, 12 h light/dark. The UC potting mix was prepared by mixing 1,200 litres of sterlised sand with 750 litres of peatmoss with the addition of calcium hydroxide (hydrated lime, 1 kg), calcium carbonate (agricultural lime, 1.8 kg) NPK fertilizer and 4 kg Osmocote per 1000 kg soil. Transgenic plantlets were grown under the same conditions. Grain harvested from plants was dried for 3 d at 37°C and then used for elemental, NA and µ-XRF studies.

### Vector construction and rice transformation

RNA was extracted from 2-week old seedlings of japonica rice cultivar Nipponbare and used for cDNA synthesis. The *OsNAS1* coding sequence was PCR amplified from cDNA with forward primer 5′ – ATGGAGGCTCAGAACCAAGAGGTCG – 3′and reverse primer 5′ – GTTAGACGGACAGCTCCTTGTTGGC – 3′ to yield a 1000 bp fragment containing the *OsNAS1* cDNA; the *OsNAS2* coding sequence was PCR amplified with forward primer 5′-ATGGAGGCTCAGAACCAAGAGGTCG – 3′ and reverse primer 5′ – ATGCACGCACTCAGACGGATAGCCT – 3′to yield a 991 bp fragment containing the *OsNAS2* cDNA; and the *OsNAS3* coding sequence was PCR amplified with forward primer 5′ – ATGACGGTGGAAGTGGAGGCGGTGA – 3′ and reverse primer 5′ – GGTGAGGTAGCAAGCGATGGAAGCA – 3′ to yield a 1072 bp fragment containing the *OsNAS3* cDNA. The three PCR fragments were cloned, separately, into the Invitrogen Gateway® Entry vector pCR8®/GW/TOPO®. Error free sequences were then recombined into a modified pMDC100 vector [23] that placed the *OsNAS* coding sequences under the control of a dual CaMV 35S promoter (Figure 1). Embryogenic nodular units arising from scutellum-derived callus were inoculated with supervirulent *Agrobacterium tumefaciens* strain AGL1 (carrying the *OsNAS* overexpression vectors) and 200 l^−1^ geneticin-resistant shoots were regenerated after nine weeks using established protocols [35]. Rooted T_0_ plantlets were transferred to the growth room in Jiffy peat pots, and moved to soil after 15 days.

### Elemental analyses of rice grain

Samples consisting of approximately 25 unpolished grain, or 35 polished grain, were analyzed by ICP-OES to determine metal concentrations. Polished grain was produced using a modified non-contaminating Kett Mill with a milling time of 2 min 30 sec, as preliminary studies with KOH staining had shown this time period to be sufficient for removal of the bran layer.

### Liquid chromatography-mass spectrometry (LC-MS)

The LC-MS method is based on published methods [36]. Four individual rice grains were obtained from each line, 20 mg of ground rice from each grain was weighed into separate Eppendorf tubes and 200 µL of EDTA solution (5 mM), containing the internal standard 2-aminobutyric acid (25 µM), was added. The EDTA was used to release any metal complexed with NA. The derivatization of nicotianamine involved mixing of 10 µL supernatant with 70 µL borate buffer (0.2 M; pH 8.8), followed by the addition of 10 µL of AQC solution (10 mM) in dry acetonitrile. The reaction mixture was then heated at 55°C for 10 min and analyzed by LC-MS. Chromatograms and mass spectra were evaluated using the MassHunter Quantitative analysis program (Agilent). Quantification was based on the external calibration curve method using the internal standard for error correction. A 1 mg/mL stock nicotianamine standard was prepared and subsequently diluted with the EDTA solution (5 mM) to prepare calibration standards in the concentration range between 2.75 – 100 µM.

### Synchrotron X-ray fluorescence spectroscopy (µ-XRF)

Thin longitudinal sections of rice grain were obtained using published methods [30]. Briefly, grains were glued to a plastic support and then sliced using a vibrating blade microtome in order to obtain a flat surface (Leica VT1000 S). A piece of Kapton polyimide film was then pressed on the surface of the sample with the blade of the microtome cutting underneath. In this way, longitudinal sections, 70 µm thick, were directly placed on Kapton tape without the need for embedding. Two longitudinal sections each of WT and OE-*OsNAS2A* grain were analyzed. µ-XRF elemental maps were collected at the X-ray Fluorescence Microscopy (XFM) beamline at the Australian Synchrotron. Whole grain elemental maps were collected at 7.5 keV using a 96-element prototype Maia detector [37]. The detector was placed perpendicular to the beam path at a distance of 20 mm and was used to collect the full spectra fluorescence signal from the sample. The samples were analysed continuously in the horizontal direction with steps of 1.25 µm in the vertical direction. The sample stage was set to a speed of 2 mm s^−1^, resulting in a pixel transit time of roughly 0.6 ms. The full XRF spectra were analysed using GeoPIXE [38], [39].

In order to map P distribution together with the distribution of the micronutrients of interest, a Vortex detector was employed as the Maia detector is unable to analyse elements lighter than K. Rectangular areas of 40×135 µm were mapped at the margin of grains with a dwell time for pixel of 1 sec. The line scans were obtained by laterally averaging the rectangular areas scanned (represented by a green box in Figure 4A).

## Supporting Information

Table S1 **Average concentrations of Fe, Zn, Mn, Cu and Ni in unpolished grain of WT and transgenic lines. **Grain samples of 3 WT, 30 OE-*OsNAS1*, 39 OE-*OsNAS2* and 24 OE-*OsNAS3* lines were analyzed by ICP-OES. Average values for each group of plants are presented as means ± standard error (S.E.) of the mean.
(DOC)
