# Supplementary material for: Constitutive Overexpression of the OsNAS Gene Family Reveals Single-Gene Strategies for Effective Iron- and Zinc-Biofortification of Rice Endosperm
Source: PLoS One. 2011 Sep 6;6(9):e24476. doi: 10.1371/journal.pone.0024476 (PMC3167849; doi:10.1371/journal.pone.0024476)
Supplement: Table S1 — Average concentrations of Fe, Zn, Mn, Cu and Ni in unpolished grain of WT and transgenic lines. Grain samples of 3 WT, 30 OE-OsNAS1, 39 OE-OsNAS2 and 24 OE-OsNAS3 lines were analyzed by ICP-OES. Average values for each group of plants are presented as means ± standard error (S.E.) of the mean. (DOC) [file pone.0024476.s001.doc]

**Table S1**.

| Genotype | Fe | Zn | Mn | Cu | Ni |
| --- | --- | --- | --- | --- | --- |
| WT | 23.3 ± 0.5 | 38.1 ± 0.3 | 14.2 ± 2.4 | 6.9 ± 1.5 | 2.3 ± 0.5 |
| OE-*OsNAS1* | 38.8 ± 1.3 | 54.5 ± 1.5 | 12.3 ± 0.3 | 8.1 ± 0.2 | 2.8 ± 0.1 |
| OE-*OsNAS2* | 42.8 ± 2.2 | 57.4 ± 2.7 | 15.8 ± 0.3 | 9.3 ± 0.3 | 2.4 ± 0.1 |
| OE-*OsNAS3* | 35.6 ± 2.4 | 48.7 ± 2.9 | 11.5 ± 0.4 | 7.7 ± 0.2 | 2.3 ± 0.1 |
